# A Phase 1b Study of Vismodegib with Pirfenidone in Patients with Idiopathic Pulmonary Fibrosis

**DOI:** 10.1007/s41030-019-0096-8

**Published:** 2019-07-19

**Authors:** Antje Prasse, Murali Ramaswamy, Shaun Mohan, Lin Pan, Andrew Kenwright, Margaret Neighbors, Paula Belloni, Peter P. LaCamera

**Affiliations:** 1 Hannover Medical School and Fraunhofer Institute for Toxicology and Experimental Medicine, Hannover, Germany; 2 PulmonIx, LLC, and Cone Health, Greensboro, NC USA; 3 Genentech, Inc., South San Francisco, CA USA; 4 St. Elizabeth’s Medical Center, Boston, MA USA

**Keywords:** Hedgehog signaling pathway, Idiopathic pulmonary fibrosis, Pirfenidone, Safety, Tolerability, Vismodegib

## Abstract

**Introduction:**

Components of the hedgehog signaling pathway are upregulated in patients with idiopathic pulmonary fibrosis (IPF). Vismodegib, a small-molecule inhibitor of hedgehog signaling, when used in combination with currently available antifibrotic therapy, may be more efficacious than antifibrotics alone. The objective of this study was to evaluate the safety and tolerability of vismodegib plus pirfenidone in patients with IPF.

**Methods:**

Twenty-one patients were enrolled in a phase 1b open-label trial to receive vismodegib 150 mg plus pirfenidone 2403 mg/day once daily. Key endpoints were safety, tolerability, and pharmacokinetics. Exploratory endpoints included change from baseline to week 24 in % predicted forced vital capacity (FVC) and University of California, San Diego Shortness of Breath Questionnaire (UCSD-SOBQ) scores, as well as pharmacodynamic changes in hedgehog biomarker C-X-C motif chemokine ligand 14 (CXCL14).

**Results:**

All patients reported at least one treatment-emergent adverse event (AE), most frequently muscle spasms (76.2%). Serious AEs were reported in 14.3% of patients; one event of dehydration was considered related to vismodegib. One patient died due to IPF progression, unrelated to either treatment. More patients discontinued vismodegib than pirfenidone (42.9% vs. 33.3%, respectively). Changes from baseline to week 24 in % predicted FVC and UCSD-SOBQ scores were within known endpoint variability. In contrast to findings in basal cell carcinoma, vismodegib had no effect on circulating CXCL14 levels.

**Conclusion:**

The safety profile was generally consistent with the known profiles of both drugs, with no new safety signals observed in this small cohort. There was no pharmacodynamic effect on CXCL14 levels. Future development of vismodegib for IPF may be limited due to tolerability issues.

**Trial Registration:**

ClinicalTrials.gov NCT02648048.

**Plain Language Summary:**

Plain language summary available for this article.

**Funding:**

F. Hoffmann-La Roche Ltd. and Genentech, Inc.

**Electronic supplementary material:**

The online version of this article (10.1007/s41030-019-0096-8) contains supplementary material, which is available to authorized users.

## Plain Language Summary

Idiopathic pulmonary fibrosis (IPF) is a progressive, irreversible, and fatal lung disease. Pirfenidone is a drug that slows disease progression in patients with IPF, but it may be more effective when combined with other drugs that target different disease pathways. This study evaluated the safety of vismodegib, an inhibitor of signaling involved in IPF, and pirfenidone in 21 patients with IPF. All patients receiving the two drugs experienced at least one side effect. The most common side effect of the two drugs was muscle spasms. Serious side effects were noted in 14% of patients, and one dehydration side effect was related to vismodegib. More patients stopped taking vismodegib than pirfenidone. Vismodegib will not be pursued as a drug for IPF because patients cannot tolerate it.

## Introduction

Idiopathic pulmonary fibrosis (IPF) is a progressive, irreversible, fatal fibrotic lung disease with a median survival of 2–5 years after diagnosis [1, 2]. Most patients with IPF are aged > 50 years, with symptoms of progressive dyspnea and nonproductive cough [1]. The clinical course of IPF in an individual patient is challenging to predict, with a variable rate of disease progression and decline [3].

IPF has a histopathologic pattern of usual interstitial pneumonia (UIP), which is characterized by patchy fibrosis interspersed with areas of normal lung appearance and regions of dense scar tissue [1]. Ongoing disease activity is hypothesized to occur at the transition zones between normal and fibrotic lung [4, 5]. A model of IPF pathogenesis suggests that alterations to the alveolar epithelium initiates mesenchymal cell expansion and differentiation as well as production of fibrogenic factors that result in excessive extracellular matrix deposition [6]. Multiple studies have investigated the role of the hedgehog signaling pathway in the pathogenesis of fibrosis [7–9]. Hedgehog signaling regulates epithelial and mesenchymal interactions in many tissues during mammalian embryogenesis [10]. Epithelial damage and subsequent dysfunctional epithelial responses may drive aberrant fibroblast activation and differentiation during fibrosis. Hedgehog signaling appears to be a strong inducer of fibrogenic responses in vitro and tissue fibrosis in vivo [9].

Components of the hedgehog pathway are upregulated in the lungs of patients with IPF [11–15]. Hedgehog signaling can contribute to fibrogenesis and is part of the epithelial–mesenchymal cross talk involving other pathways active in IPF, such as transforming growth factor *β*, C-X-C motif chemokine receptor type 4, and interleukin 13, resulting in increased myofibroblast differentiation, extracellular matrix production, motility, and survival. Studies comparing lung samples from patients with IPF and control individuals have demonstrated that expression of hedgehog ligands in type II alveolar and bronchiolar epithelial cells is upregulated under conditions of disease [15]. Also, expression of hedgehog target genes, such as C-X-C motif chemokine ligand 14 (*CXCL14*), is increased in lung fibroblasts and detected at elevated levels in lungs of patients with IPF [12, 13, 15–17].

Vismodegib, a small-molecule inhibitor of the hedgehog signaling pathway, binds to and inhibits smoothened, a transmembrane protein involved in canonical hedgehog signaling [18]. Vismodegib received approval by the US Food and Drug Administration in 2012 for the treatment of adults with metastatic basal cell carcinoma (BCC) or with locally advanced BCC that had recurred following surgery or who were not candidates for surgery or radiation therapy [19]. In the pivotal registration study (SHH4476g) in metastatic or locally advanced BCC, 30% of patients (95% CI 16–49%; *p* = 0.001) achieved the primary efficacy outcome of an objective response as assessed by an independent review facility [18]. The median duration of treatment was 9.8 months at the time of the primary analysis [18]. Treatment-emergent adverse events (TEAEs), defined as any new AE reported or any worsening of an existing condition reported on or after the first dose of study drug, occurred at a rate of ≥ 10% and included decreased appetite, dysgeusia, ageusia, nausea, diarrhea, constipation, vomiting, alopecia, muscle spasms, amenorrhea, weight loss, and fatigue [18].

Previous reports have shown that circulating CXCL14 protein levels are significantly higher in plasma from patients with IPF than in controls, and circulating CXCL14 levels are significantly reduced upon vismodegib treatment in patients with cancer, indicating that circulating CXCL14 levels can reflect hedgehog pathway signaling in some settings [16]. However, noncanonical hedgehog signaling can also occur [14], and it is unclear whether hedgehog pathway activation in IPF is predominantly canonical. Also, there are several other pathways active in IPF with potential to contribute to CXCL14 levels, including signaling by WNT, hypoxia-inducible factor 1-alpha, vascular endothelial growth factor, epidermal growth factor, and oxidative stress [17]. Therefore, CXCL14 was explored in this study to determine whether it was suitable as an indicator of pharmacological activity for vismodegib in IPF.

Pirfenidone is one of two approved oral antifibrotic therapies for the treatment of IPF [20–22]. Although its exact mechanism of action has not been fully established, pirfenidone has exhibited anti-inflammatory, antioxidant, and antifibrotic properties in some studies, including reductions in levels of tumor necrosis factor *α*, transforming growth factor *β*, and more recently, zinc finger protein GLI2, a component of the hedgehog pathway [23–25]. Notably, these studies were not conducted using physiologically relevant concentrations of pirfenidone. Clinically, pirfenidone has slowed disease progression of IPF as measured by changes in percent predicted forced vital capacity (% predicted FVC) and reduced the risk of death as demonstrated in pooled analyses of three phase 3 pivotal trials: ASCEND (Study 016, NCT01366209) and the CAPACITY studies (Study 004, NCT00287716 and Study 006, NCT00287729]) [20, 21, 26, 27]. In patients with IPF, pirfenidone has been generally well tolerated, with manageable AEs, most notably gastrointestinal and skin-related [20, 21, 26, 28]. Previous studies have shown that pirfenidone does not modulate CXCL14 levels in patients providing biomarker samples from these trials [29].

Targeting multiple pathways involved in the pathogenesis of lung fibrosis with combination therapy of pirfenidone and vismodegib may maximize the efficacy of the currently available standard of care for patients with IPF. The primary objective of this study was to evaluate the safety and tolerability of vismodegib in combination with pirfenidone in patients with IPF.

## Methods

### Study Design

**Fig. 1:**
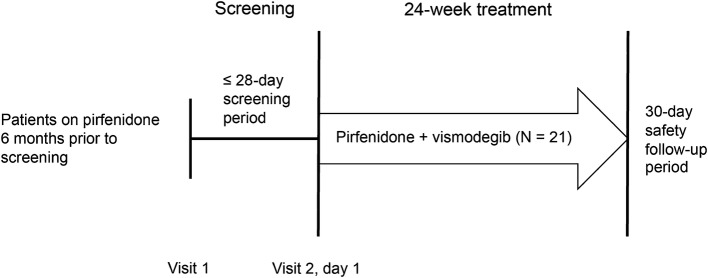
Study design

ISLAND2 was a single-arm, multicenter, open-label, phase 1b trial that assessed the safety and tolerability of vismodegib in combination with pirfenidone in patients with IPF (NCT02648048). Patients who provided written informed consent entered a screening period of up to 28 days to establish study criteria (visit 1) (Fig. 1). Eligible patients were then enrolled in a single treatment arm for oral administration of vismodegib 150 mg once daily plus pirfenidone ≤ 801 mg/day three times daily with food.

The treatment duration was 24 weeks, with the first dose of vismodegib administered at the enrollment visit (visit 2, day 1). All patients who completed therapy, as well as those who discontinued study treatment early, were asked to complete a 30-day safety follow-up period during which only pirfenidone was administered. The maximum total study duration for any patient was approximately 28 weeks from the first administration of vismodegib. No dose reductions of vismodegib were permitted based on previous studies in BCC [18]. Treatment with vismodegib could be interrupted for up to 8 weeks for evaluation of an intolerable toxicity; the original dose was maintained upon restart of treatment.

All procedures followed were in accordance with the ethical standards of the responsible committee on human experimentation (institutional and national) and with the Helsinki Declaration of 1964, as revised in 2013. All investigators obtained institutional review board (IRB) approval for the investigation either through the central IRB located in Seattle, WA, or via local IRBs (Table S1 in the Electronic Supplementary Material), and all patients provided informed consent. The ClinicalTrials.gov registration number for this study is NCT02648048.

### Patients

Patients who were aged 40–80 years at visit 1, had clinical symptoms consistent with IPF for ≥ 12 months, and had a diagnosis of UIP or IPF by high-resolution computed tomography (HRCT) and/or surgical lung biopsy within 5 years (Table S2 in the Electronic Supplementary Material) were enrolled [1]. A central review assessment of HRCT was performed during the screening period or ≤ 12 months prior to the start of screening. Patients had to be receiving stable pirfenidone (≥ 1602 mg/day) for ≥ 8 weeks prior to randomization, without adverse drug reactions, tolerating pirfenidone 1602–2403 mg/day for ≥ 24 weeks prior to and during screening. Additional criteria included % predicted FVC ≥ 50% and ≤ 100% at screening; percent predicted diffusing capacity for carbon monoxide (DLco) ≥ 30% and ≤ 90% at screening; adequate hematopoietic, renal, and liver function; and absolute avoidance of pregnancy.

Key exclusion criteria were known hypersensitivity to any components of the study drugs or the drugs themselves; prior treatment with vismodegib or any hedgehog pathway inhibitor; evidence of other known causes of interstitial lung disease; hospitalization due to an IPF exacerbation ≤ 4 weeks prior to or during screening; lung transplant expected within 6 months of screening; evidence of clinically significant lung disease other than IPF; known current malignant neoplasm or current evaluation for a potential malignancy; tobacco smoking within 3 months of screening or unwillingness to avoid smoking throughout the study; and any condition that, as assessed by the investigator, might be significantly exacerbated by the known AEs associated with pirfenidone.

### Assessments

Disease-specific assessments included spirometry, DLco, HRCT, surgical lung biopsy, and University of California, San Diego Shortness of Breath Questionnaire (UCSD-SOBQ) [30, 31]. Safety assessments included TEAEs, AEs of special interest (AESIs), clinical laboratory tests, and vital signs. Vismodegib-specific AESIs included muscle spasms and drug-induced liver injury, for which monitoring was conducted for elevated alanine aminotransferase or aspartate aminotransferase levels, with either elevated bilirubin levels or clinical jaundice. Pirfenidone-specific AESIs included gastrointestinal and skin disorders, elevated liver enzyme values, and photosensitivity reaction or rash.

Serum and plasma samples were collected during screening, at baseline, and throughout the study for pharmacokinetics, exploratory analyses, and laboratory assessments. Single predose trough plasma samples were collected from all patients at weeks 4, 12, and 24 and at the 30-day safety follow-up visit following the last dose of vismodegib. The total and unbound concentrations of vismodegib in plasma were quantified using a validated solid-phase extraction and liquid chromatography-tandem mass spectrometry method [32].

CXCL14 was the primary hedgehog pathway biomarker investigated to evaluate vismodegib pharmacodynamic activity and was measured in plasma using a prototype Elecsys^®^ platform (Cobas e; Roche Diagnostics, Penzberg, Germany) [16].

### Study Objectives

The primary objective was to evaluate the safety and tolerability of the vismodegib and pirfenidone combination. The pharmacokinetics objective was to evaluate the pharmacokinetics of vismodegib when administered in combination with pirfenidone. The exploratory objectives were to investigate the efficacy of vismodegib in combination with pirfenidone based on change from baseline to week 24 in % predicted FVC and in dyspnea, as measured by UCSD-SOBQ score, as well as pharmacodynamic change in CXCL14 levels.

### Statistical Analyses

The analysis population comprised the safety population, intent-to-treat population (ITT), pharmacokinetics population, and pharmacodynamics population. The safety population consisted of all enrolled patients who received at least one dose of any study treatment and had at least one post-dose safety assessment. The ITT population consisted of all enrolled patients who received at least one dose of any study treatment. The pharmacokinetics population included all safety-evaluable patients with a post-dose pharmacokinetics sample. The pharmacodynamics population included all patients with non-missing biomarker data available at baseline and at least one non-missing post-baseline measurement in addition to meeting dosing criteria (discontinued vismodegib < 3 consecutive days prior to sample collection or redosed continuously > 11 days after a lapse in dosing for any reason).

The study was not powered, and no hypothesis testing was performed due to the exploratory nature of the study. A sample size of approximately 20 patients was determined based on safety and clinical considerations. All data were summarized using descriptive statistics. The severity of AEs was graded according to the National Cancer Institute Common Terminology Criteria for Adverse Events version 4.0 [33]. Log-transformed biomarker data were evaluated by a linear mixed-effects model, with visit variable as a fixed effect and patient as a random effect to determine whether on-study biomarker levels at weeks 4, 12, and 24 differed from baseline, with two-sided 95% confidence intervals.

## Results

### Patient Disposition

**Fig. 2:**
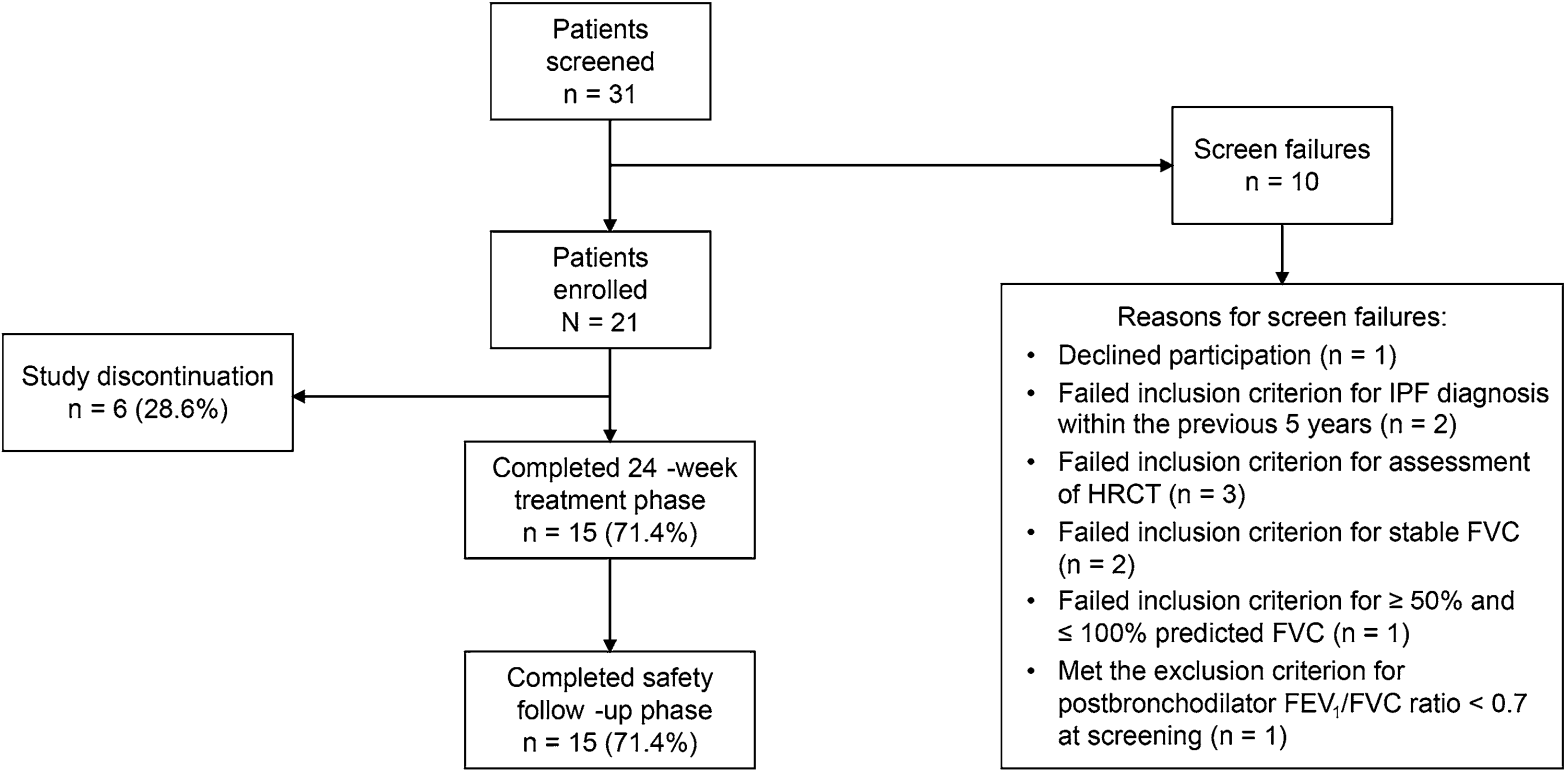
Patient disposition. *FEV*_1_ forced expiratory volume in 1 s, *FVC* forced vital capacity, *HRCT* high-resolution computed tomography*, IPF* idiopathic pulmonary fibrosis

A total of 31 patients with IPF receiving background pirfenidone were screened, of whom 10 were deemed ineligible (Fig. 2). Overall, 21 patients were enrolled between January 15, 2016, and May 18, 2016, at 16 sites in the United States and one site in Germany (*n* = 5). Fifteen patients (71.4%) completed the 24-week treatment phase and the safety follow-up period. Six patients (28.6%) discontinued the study during the 24-week treatment period (Table 1). Four patients (19.0%) discontinued due to AEs, one patient (4.8%) withdrew, and one patient (4.8%) discontinued for other reasons. Nine patients (42.9%) stopped vismodegib treatment during the study, of whom seven (33.3%) discontinued all treatment (Table 2). The primary reason for discontinuation of study drug was a TEAE associated with vismodegib (six of nine patients) or pirfenidone (one of seven patients).

**Table 1:** Patient disposition through week 24

Status, *n* (%)	Vismodegib 150 mg/day + pirfenidone ≤ 2403 mg/day (*N* = 21)
Completed 24-week treatment phase	15 (71.4)
Completed safety follow-up	15 (71.4)
Discontinued 24-week treatment phase	6 (28.6)
Adverse event	4 (19.0)
Withdrawal by patient	1 (4.8)
Other	1 (4.8)

**Table 2:** Discontinuations of vismodegib and pirfenidone through week 24 (*N* = 21)

Status, *n* (%)	Vismodegib 150 mg/day	Pirfenidone ≤ 2403 mg/day
Study drug discontinuations	9 (42.9)^a^	7 (33.3)
AEs	6 (28.6)	1 (4.8)
Death	0	0
Other	1 (4.8)	1 (4.8)
Physician decision	0	0
Protocol violation	0	0
Withdrawal by patient	2 (9.5)	5 (23.8)

*AE* adverse event, *SAE* serious adverse event

^a^Four of nine patients discontinued vismodegib due to muscle spasm AEs, and one of nine patients discontinued vismodegib due to a dehydration SAE

### Patients

All 21 enrolled patients received at least one dose of study drug and were included in the safety and intent-to-treat populations. Twenty patients were included in the pharmacokinetics analysis population. By the end of week 24, 12 patients remained in the intent-to-treat population.

The mean (standard deviation [SD]) age was 70.6 (6.8) years, and most patients were male and white (90.5% each) (Table 3). Eighteen patients (85.7%) had a history of smoking. The mean (SD) IPF duration was 2.45 (1.37) years. The most frequent concurrent medical conditions reported by patients were hypertension (*n* = 12 [57.1%]), hyperlipidemia (*n* = 8 [38.1%]), and gastroesophageal reflux disease (*n* = 7 [33.3%]). The most commonly reported concomitant medications were proton pump inhibitors (*n* = 18 [85.7%]), salicylates (*n* = 13 [61.9%]), and statins (*n* = 13 [61.9%]). Four patients (19.0%) reported prior pirfenidone use.

**Table 3:** Baseline demographics and clinical characteristics

Characteristic	Vismodegib 150 mg/day + pirfenidone ≤ 2403 mg/day (*N* = 21)
Age, mean (SD), years	70.6 (6.8)
Male, *n* (%)	19 (90.5)
White, *n* (%)	19 (90.5)
BMI, mean (SD), kg/m^2^	28.93 (3.27)
Time from IPF diagnosis, mean (SD), years	2.45 (1.37)
Smoking status, *n* (%)	
Never	3 (14.3)
Former	18 (85.7)
Current	0
FVC, mean (SD), % predicted	67.38 (13.29)
DLco, mean (SD), % predicted^a^	62.79 (24.13)
UCSD-SOBQ score, mean (SD)	48.62 (22.49)

*BMI* body mass index, *DLco* diffusing capacity for carbon monoxide, *FVC* forced vital capacity, *IPF* idiopathic pulmonary fibrosis, *SD* standard deviation, *UCSD*-*SOBQ* University of California, San Diego Shortness of Breath Questionnaire

^a^Corrected for alveolar volume and hemoglobin

### Safety

All 21 patients experienced at least one TEAE during the study, and a total of 135 TEAEs were reported (Table 4), most of which were considered mild or moderate in intensity by the investigator. The most frequent TEAEs were muscle spasms (*n* = 16 [76.2%]) and dysgeusia (*n* = 13 [61.9%]) (Table 5; Table S3 in the Electronic Supplementary Material). One AE of decreased appetite of life-threatening intensity occurred and was considered by the investigator to be related to vismodegib.

**Table 4:** Safety overview

	Vismodegib 150 mg/day + pirfenidone ≤ 2403 mg/day (*N* = 21)
Patients with ≥ 1 TEAE, *n* (%)	21 (100)
Total number of TEAEs	135
TEAE leading to withdrawal from study, *n* (%)	4 (19.0)
Patients with ≥ 1 SAE, *n* (%)	3 (14.3)
Total number of SAEs	5
Related SAE, *n* (%)^a^	1 (4.8)
Deaths, *n* (%)^b^	1 (4.8)
Patients with ≥ 1 AESI	
Muscle spasms, *n* (%)	16 (76.2)
Total number of muscle spasm events	28
Infections, *n* (%)	7 (33.3)
Total number of infections	7

*AESI* adverse event of special interest, *IPF* idiopathic pulmonary fibrosis, *SAE* serious adverse event, *TEAE* treatment-emergent adverse event

^a^SAE dehydration related to vismodegib

^b^Cause of death reported as IPF

**Table 5:** Treatment-emergent adverse events in ≥ 10% of patients in the safety-evaluable population

TEAE, *n* (%)	Vismodegib 150 mg/day + pirfenidone ≤ 2403 mg/day (*N* = 21)
Total	21 (100)
Muscle spasms	16 (76.2)
Dysgeusia	13 (61.9)
Alopecia	7 (33.3)
Weight loss	7 (33.3)
Decreased appetite	6 (28.6)
IPF	3 (14.3)
Nausea	3 (14.3)

*IPF* idiopathic pulmonary fibrosis, *TEAE* treatment-emergent adverse event

A total of five treatment-emergent serious AEs (TESAEs) were reported in three patients (one event each of parainfluenza virus infection, dehydration, and pulmonary embolism; two events of IPF progression). The TESAE of dehydration was considered related to vismodegib by the investigator (Table 4).

Sixteen patients (76.2%) reported at least one AESI of muscle spasms, all considered vismodegib-related (28 total events). Most muscle spasms occurred in both arms and both legs, started early during treatment at week 4, and were of mild or moderate intensity; however, four of nine patients who discontinued vismodegib reported reasons related to muscle spasms. Infection AESIs were reported by seven patients (33.3%) and comprised respiratory tract infections (two patients), and one each of bronchitis, pneumonia, rhinitis, influenza, and parainfluenza. One patient died due to progression of IPF after completion of the last dose of drug administration, but this event was considered unrelated to either vismodegib or pirfenidone.

Patients received vismodegib treatment for 5–26 weeks, with 11 of 21 patients (52%) receiving the protocol-defined 24 weeks of treatment. The mean (SD) treatment duration was 19.2 (6.6) and 22.9 (6.6) weeks for vismodegib and pirfenidone, respectively (Table S4 in the Electronic Supplementary Material). One patient had a treatment holiday (i.e., missed treatment for ≥ 7 days) for 34 days. Another two patients had vismodegib dosing interrupted for < 7 days.

### Pharmacokinetics

The observed total and free trough plasma concentrations of vismodegib following daily oral administration of vismodegib plus pirfenidone were relatively constant at steady state over time (range 7–9 μg/mL and 0.07–0.09 μg/mL, respectively) (Table S5 in the Electronic Supplementary Material). These levels were consistent with vismodegib exposures in patients with cancer in the previous studies of vismodegib using the same dosing regimen [32, 34–37].

### Pharmacodynamics

**Fig. 3:**
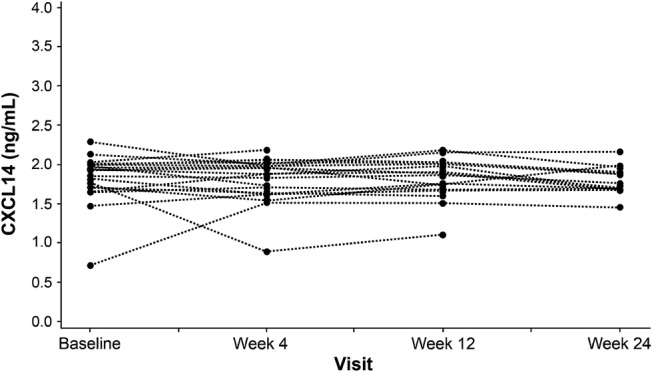
Individual CXCL14 levels. Plasma CXCL14 levels in individuals over time after initiation of vismodegib treatment. Baseline is the patient’s last observation prior to initiation of vismodegib. Dashed lines connect matched longitudinal samples from individual patients. *CXCL14* C-X-C motif chemokine ligand 14

CXCL14 baseline levels were comparable to those in other IPF studies (data not shown). No meaningful change from baseline was observed at weeks 4, 12, and 24 after vismodegib treatment (Fig. 3).

### Exploratory Efficacy

**Fig. 4:**
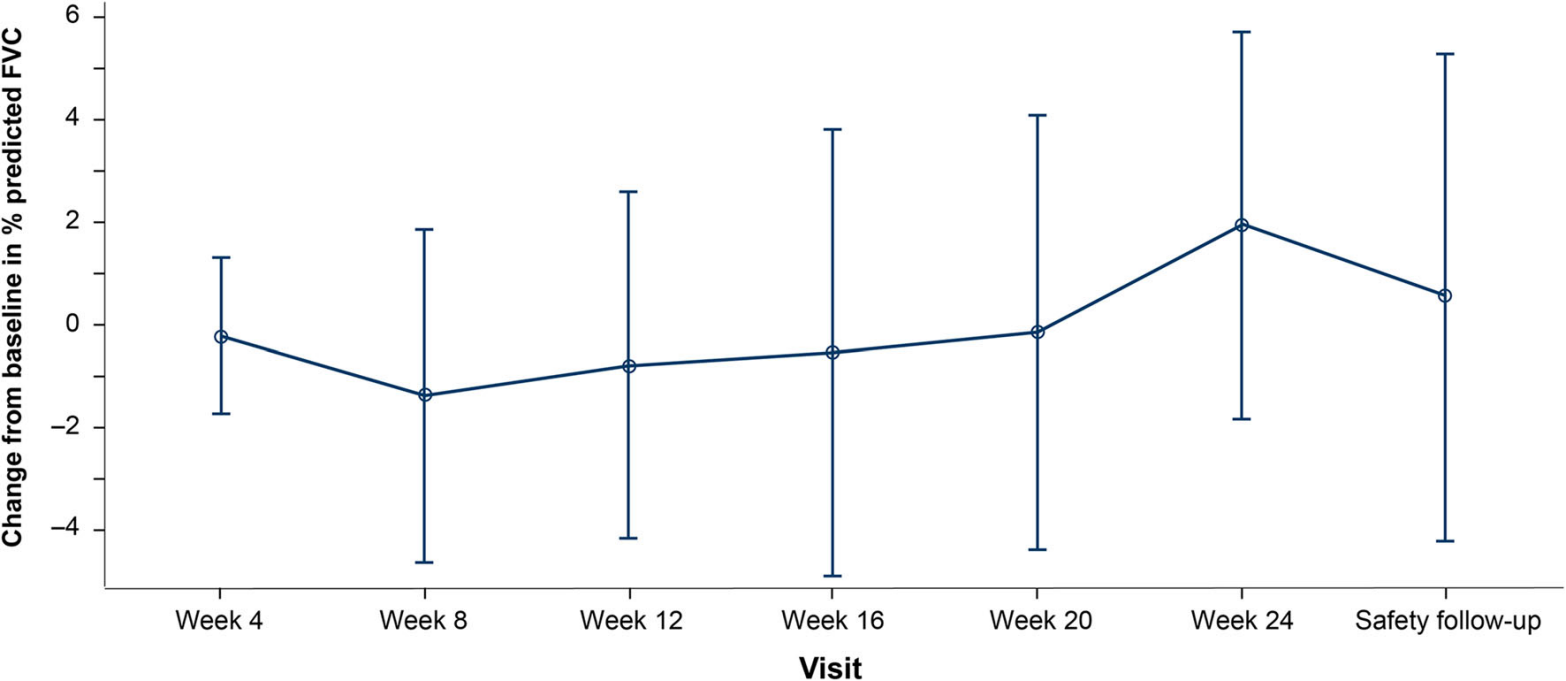
Mean change from baseline % predicted FVC score for the 12 patients in the ITT population. Within a given visit, the best (maximum) result for the parameter of interest was used if it was from an acceptable blow as assessed by the over-reader. Baseline is the patient’s last observation prior to initiation of vismodegib. If multiple records were collected during the same visit, then the last record within that last visit was used for the analysis. *FVC* forced vital capacity, *ITT* intent to treat

The mean (SD) change from baseline in  % predicted FVC to week 24 was 2.0% (5.94%). Changes in % predicted FVC from baseline for the 12 patients in the ITT population were within the known inherent variability for this endpoint (Fig. 4) [38–40].

A positive trend in dyspnea as assessed by UCSD-SOBQ was observed in this small cohort of patients who completed the trial. The mean (SD) changes from baseline in UCSD-SOBQ score at weeks 12 (*n* = 19) and 24 (*n* = 12) were 2.00 (20.29) and −4.58 (17.05), respectively. However, due to the large number of patient discontinuations (12 patients remaining by week 24 for the exploratory analysis), the observed change was lower than the expected minimum clinically important difference of 8 in an IPF population [41].

## Discussion

The rationale for investigating the combination of vismodegib with pirfenidone was to increase the efficacy of currently available antifibrotic therapies in patients with IPF. In this phase 1b trial, the overall safety profiles observed with both study drugs were consistent with the known safety profiles for vismodegib and pirfenidone. Rates of TEAEs based on preferred terms in the System Organ Class groups of infection as well as respiratory, thoracic, and mediastinal disorders were evenly distributed. No new safety signals were observed, and the incidence of SAEs was low. There was one death due to IPF disease progression.

The treatment discontinuation rate for vismodegib (42.9%) was moderately higher than that for pirfenidone (33.3%) and higher than those observed in other pirfenidone monotherapy and combination studies [20, 21, 42]. The most frequently reported TEAEs were muscle spasms (76.2%) and dysgeusia (61.9%), which may have contributed to the observed elevated discontinuation rate. The rates of muscle spasms and dysgeusia were consistent with the rates observed in the phase 1b/2 registration trials in BCC (68% and 51%, respectively) [18]. This AE profile associated with vismodegib treatment is likely the mechanism based on similar AEs that have been reported for another inhibitor of hedgehog signaling, sonidegib [43]. In a phase 2 study of sonidegib, muscle spasms were the most commonly reported AE in 49% and 67% of patients in the 200 mg and 800 mg groups, respectively, and the most common AE resulting in treatment discontinuation [43].

The plasma concentrations of vismodegib in patients with IPF were consistent with those observed previously in oncology studies in which the drug was given in the same dosing regimen [34–37].

There is evidence from patients with cancer that plasma CXCL14 levels are sensitive to modulation by vismodegib, yet no pharmacodynamic effect on this biomarker was observed in our study of patients with IPF [16]. It is unlikely that this result is indicative of failure to engage the receptor, because the safety findings are considered to be target-related. More likely, this finding indicates that the systemic levels of this hedgehog pathway biomarker were insensitive to vismodegib and may suggest that noncanonical, smoothened-independent signaling contributes more to hedgehog pathway activity in patients with IPF. However, it could also indicate that CXCL14 is not suitable for monitoring hedgehog pathway activity in IPF because other pathways active in IPF have a greater influence on circulating CXCL14 levels.

In a 24-week study of patients with IPF treated with pirfenidone, a decrease in FVC (≈35–70 mL) would have been expected [38]. In this study, however, a slight increase in FVC was observed. Furthermore, after vismodegib discontinuation (24 weeks), FVC values started to decrease again. These FVC changes were likely not meaningful, particularly given the small sample size and heterogeneous intraindividual variability in treatment response observed in real-world patient cohorts [39, 44]. Based on the lack of a comparator arm, short study duration, small number of patients, and the high discontinuation rate, it is difficult to draw any conclusions from these findings. However, the magnitude of the effect of combination therapy with pirfenidone and vismodegib on FVC was within a range similar to that observed in a trial that assessed combination therapy with pirfenidone and nintedanib for 6 months [42].

Limitations of the study include a small sample size, high discontinuation rate, and the lack of a comparator arm and statistical powering. The observed change from baseline in FVC and UCSD-SOBQ at week 24 were within the known variability inherent in these endpoints.

## Conclusion

No new safety signals were identified with the combination of pirfenidone and vismodegib in patients with IPF. The development of vismodegib for treatment of patients with IPF has been discontinued due to the tolerability issues observed in this study. Future studies will be needed to determine how hedgehog signaling can be efficiently inhibited with fewer systemic AEs (e.g., different target or mode of administration).

## Electronic Supplementary Material

Below is the link to the electronic supplementary material.

Supplementary material 1 (PDF 246 kb)
