# Supplementary material for: A Phase 1b Study of Vismodegib with Pirfenidone in Patients with Idiopathic Pulmonary Fibrosis
Source: Pulm Ther. 2019 Jul 19;5(2):151–63. doi: 10.1007/s41030-019-0096-8 (PMC6967289; doi:10.1007/s41030-019-0096-8)
Supplement: Supplementary file 1 — Supplementary material 1 (PDF 246 kb) [file 41030_2019_96_MOESM1_ESM.pdf]

A Phase 1b Study of Vismodegib With Pirfenidone in Patients With Idiopathic Pulmonary Fibrosis

Antje Prasse, Murali Ramaswamy, Shaun Mohan, Lin Pan, Andrew Kenwright, Margaret Neighbors, Paula Belloni, Peter P. LaCamera

SUPPLEMENTARY MATERIAL

Additional file

Additional file 1: Table S1. Ethics committees and IRB details. **Table S2.** Combined HRCT and SLB eligibility assessment. **Table S3.** Treatment-emergent adverse events in the safety-evaluable population. **Table S4.** Exposure to vismodegib and pirfenidone. **Table S5.** Vismodegib pharmacokinetics.

Table S1. Ethics committees and IRB details.

**Last Name**	**First Name**	**Ethics Committee/IRB**	**Address**
Central IRB			
		Quorum IRB (7 sites)	1501 Fourth Avenue, Suite 800 Seattle, WA 98101
Local IRB			
Cary	Jeff	WIRB	1019 39th Avenue SE Puyallup, WA, 98374
Chang	Jacqueline	Scripps Health IRB	11025 N. Torrey Pines Rd., Suite 200 La Jolla, CA 92037
LaCamera	Peter	Steward St. Elizabeth's Medical Center IRB	736 Cambridge St Boston, MA 02135
Lasky	Joseph A	Tulane University Human Research Protection Office	1430 Tulane Avenue TW-36 New Orleans, LA 70112
Morrow	Lee	Creighton University IRB	2500 California Plaza Omaha, NE 68178
Ramaswamy	Murali	Cone Health IRB	1200 North Elm Street Room 1956 Greenboro, NC

			27401
Roman	Jesse	University of Louisville Human Subject Protection	501 E. Broadway<br>MedCenter One,<br>Suite 200<br>Louisville, KY 40202

IRB Institutional Review Board.

Table S2. Combined HRCT and SLB eligibility assessment

	SLB Assessment					
**HRCT Assessment**	**Definite UIP**	**Probable UIP**	**Possible UIP**	**Non-Classifiable Fibrosis**	**Inconsistent With UIP**	**No SLB**
Definite UIP	Eligible	Eligible	Eligible	Eligible	Not eligible	Eligible
Possible UIP	Eligible	Eligible	Eligible	Eligible	Not eligible	Eligible
Inconsistent with UIP	Not eligible	Not eligible	Not eligible	Not eligible	Not eligible	Not eligible

HRCT high-resolution computed tomography, *SLB* surgical lung biopsy, *UIP* usual interstitial pneumonia

Table S3. Treatment-emergent adverse events in the safety-evaluable population

**TEAE, n (%)**	**Vismodegib 150 mg/day + Pirfenidone ≤ 2403 mg/day (N = 21)**
Total patients with ≥ 1 TEAE, n (%)	21 (100)
Muscle spasms	16 (76.2)
Dysgeusia	13 (61.9)
Alopecia	7 (33.3)
Weight decreased	7 (33.3)
Decreased appetite	6 (28.6)
IPF	3 (14.3)
Nausea	3 (14.3)
Blood creatine phosphokinase increased	2 (9.5)
Constipation	2 (9.5)
Diarrhea	2 (9.5)
Dry skin	2 (9.5)
Fatigue	2 (9.5)
Hair growth abnormal	2 (9.5)
Photosensitivity reaction	2 (9.5)
Rash	2 (9.5)
Abdominal pain	1 (4.8)
Abdominal pain upper	1 (4.8)
Abnormal loss of weight	1 (4.8)
Arthralgia	1 (4.8)
Asthenia	1 (4.8)

Bronchitis	1 (4.8)
Chest pain	1 (4.8)
Chills	1 (4.8)
Cough	1 (4.8)
Dehydration	1 (4.8)
Dry mouth	1 (4.8)
Dyspnea	1 (4.8)
Ear pain	1 (4.8)
Flatulence	1 (4.8)
FVC decreased	1 (4.8)
Gastroesophageal reflux disease	1 (4.8)
Gout	1 (4.8)
Headache	1 (4.8)
Hemoglobin increased	1 (4.8)
Hemoptysis	1 (4.8)
Hypoacusis	1 (4.8)
Hypotension	1 (4.8)
Hypoxia	1 (4.8)
Increased appetite	1 (4.8)
Increased viscosity of upper respiratory secretion	1 (4.8)
Influenza	1 (4.8)
Iron deficiencies	1 (4.8)
Laceration	1 (4.8)
Lip blister	1 (4.8)
Liver function test increased	1 (4.8)

Loss of libido	1 (4.8)
Lung infection	1 (4.8)
Memory impairment	1 (4.8)
Parainfluenza virus infection	1 (4.8)
Pneumonia	1 (4.8)
Pruritus	1 (4.8)
Pulmonary embolism	1 (4.8)
Pyrexia	1 (4.8)
Respiratory tract infection	1 (4.8)
Rhinitis	1 (4.8)
Rhinorrhea	1 (4.8)
Skin hemorrhage	1 (4.8)
Skin injury	1 (4.8)
Stomatitis	1 (4.8)
Toothache	1 (4.8)
Upper respiratory tract infection	1 (4.8)
Ventricular extrasystoles	1 (4.8)
Vomiting	1 (4.8)

FVC forced vital capacity, *IPF* idiopathic pulmonary fibrosis, *TEAE* treatment-emergent adverse event

Table S4. Exposure to vismodegib and pirfenidone

	**Vismodegib 150 mg/day + Pirfenidone ≤ 2403 mg/day (N = 21)**
**Vismodegib 150 mg/day**	
Treatment duration, mean (SD), weeks	19.2 (6.6)
Dose intensity, mean (SD), %	77.64 (27.07)
**Pirfenidone ≤ 2403 mg/day**	
Treatment duration, mean (SD), weeks	22.9 (6.6)

SD standard deviation

Table S5. Vismodegib pharmacokinetics

Vismodegib Concentration, Mean (SD), µg/mL	C_{min}, Week 4 (n = 20)	C_{min}, Week 12 (n = 20)	C_{min}, Week 24 (n = 15)	SFU, Week 30 (n = 15)
Total	9.39 (2.32)	8.29 (3.27)	7.09 (4.57)	1.18 (1.65)
Free	0.09 (0.04)	0.08 (0.05)	0.07 (0.06)	0.01 (0.01)[a]

C_{min} concentration at 24 ± 0.17 hours following the most recent dose, *SD* standard deviation,

SFU plasma vismodegib concentration at Day 30 after last dose

[a] n = 12
